# Aging, HIV, and Vascular Health: Implications for Black Men’s Brain Health

**DOI:** 10.1093/geroni/igaf122.1303

**Published:** 2025-12-31

**Authors:** Raymond Jones

**Affiliations:** The University of Alabama at Birmingham, Birmingham, Alabama, United States

## Abstract

As Black men with HIV age, they face compounded risk of neurocognitive decline and cardiovascular disease, driven by chronic inflammation, vascular dysfunction, and structural inequities. HIV accelerates biological aging, increasing cerebrovascular injury and cognitive impairment. Hypertension, diabetes, and other risk factors compound these effects, disproportionately affecting Black men due to barriers to healthcare access. Emerging work underscores the role of endothelial dysfunction and cerebrovascular injury in HIV, aging, and cognitive impairment. Given the intersectionality of these conditions, addressing brain and health in aging Black men with HIV requires a multidisciplinary, culturally tailored approach. Lifestyle modifications – physical activity and diet – show promise in reducing neurovascular damage. Community-based interventions, culturally tailored health education, and improved access to care are critical for promoting healthy aging. This presentation will highlight the need for interdisciplinary, equity-focused approaches to preserve brain health in aging Black men with HIV and reduce disparities in cognitive and cardiovascular outcomes.

